# Locally Invasive Pigmented Villonodular Synovitis of the Wrist Treated by Arthroscopic Resection and Bone Grafting

**DOI:** 10.1111/os.13921

**Published:** 2023-10-25

**Authors:** Rebecca Qian Ru Lim, Xing Shuai Gao, Bo Liu

**Affiliations:** ^1^ Department of Hand & Reconstructive Microsurgery Singapore General Hospital Singapore; ^2^ Department of Orthopaedic Surgery Zhongshan Ji Shui Tan Orthopaedic Hospital Guangdong China; ^3^ Department of Hand Surgery Beijing Ji Shui Tan Hospital Beijing China

**Keywords:** Pigmented Villonodular Synovitis, Scaphoid, Wrist Arthroscopy

## Abstract

Pigmented villonodular synovitis (PVNS) is a benign but locally aggressive neoplasm that can affect tendon sheath, bursae, or joint. The wrist joint however is uncommonly involved and here we present a case of chronic monoarticular joint pain and swelling in a healthcare professional that was later histologically verified to be PVNS of the radiocarpal joint. The patient had a magnetic resonance imaging (MRI) performed prior to surgery which showed a locally invasive bony tumor of the scaphoid. He subsequently underwent a wrist arthroscopic evaluation and resection with bone grafting as the index surgery and made an uneventful postoperative recovery. This is a novel technique to address PVNS of the wrist as these cases are usually managed using open procedures which can lead to additional scarring and disrupt the blood supply of the joint capsule.

## Graphical abstract

We present an uncommon case of wrist pigmented villonodular synovitis (PVNS) that was painful with aggressive local bony invasion to the scaphoid. The patient underwent a novel surgery using wrist arthroscopic evaluation and resection with bone grafting in one sitting and made an uneventful postoperative recovery.

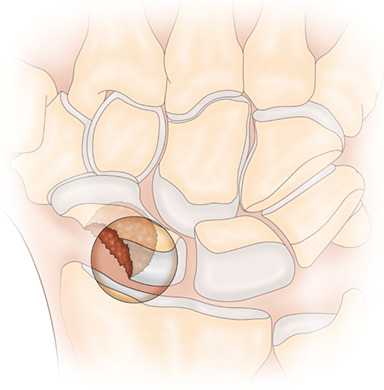

## Introduction

PVNS is a rare proliferative disorder that involves tendon sheaths and synovial joints.^1^ Although they can be locally aggressive, they are not known to have malignant potential or metastasize. The etiology of this condition remains idiopathic and clinical findings vary based on the joint involved, tumor size, and extent of extra‐articular involvement.

Most patients report a period of progressive pain, followed by diminished range of motion, and a possible mass effect.^1^ This can be debilitating and affect the quality of life and livelihood of patients afflicted with this condition.

Surgical excision remains the treatment of choice for this proliferative disorder, and it is important to achieve complete resection as recurrence rates can range to be as high as 45% for diffuse PVNS of the knee joint.^2^ The resection is predominantly done as an open technique, and this can lead to joint stiffness and compromise the local blood supply of the operated region.

With the increasing interest and advent of minimally invasive procedures, we present to the best of our knowledge, the first case report of a radiocarpal joint PVNS that was treated using wrist arthroscopic resection and artificial bone grafting. We believe that this will be of interest as it is a minimally invasive procedure that can better preserve the local blood supply of the joint, reduce injury to surrounding soft tissue structures, and at the same time provide good magnification of the surgical site for complete resection of the lesion.

## Case Report

A 28‐year‐old dentist presented with recent exacerbation of a 6‐year history of mechanical non‐ traumatic radial sided wrist pain. He was also concerned about a painless but slowly growing bony protuberance over the site of tenderness. His symptoms spontaneously resolved with rest in the initial years but the intensity and duration of the painful episodes in the past 6 months had significantly increased which prompted him to undergo a formal clinical evaluation.

This study was approved by the Ethics Committee at the Zhongshan Jishuitan Hospital (V20230506).

The patient was noted to have significant pain and swelling over the radial aspect of the wrist with a bony growth located over the scaphoid tubercle and anatomical snuffbox region. Range of motion and grip strength as compared to the normal contralateral upper limb was >50% decreased. Neurological examination of both upper limbs was normal.

**FIGURE 1:**
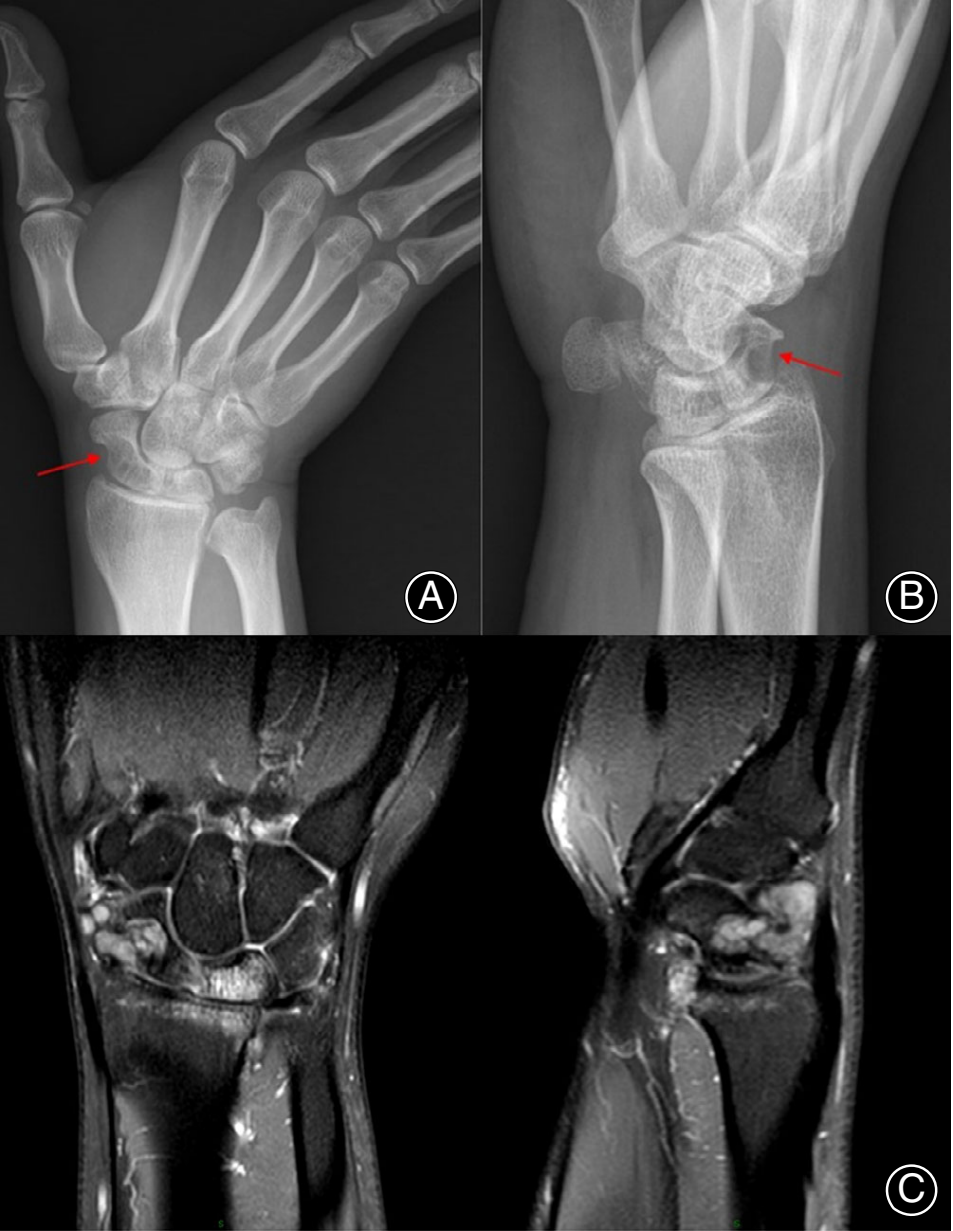
(A. B) X‐rays of the right wrist showing scaphoid deformity with radial aspect concavity suggestive of a local compressive effect (red arrows). (C) MRI of the right wrist showing the infiltrative bony tumor of the scaphoid.

The X‐rays of the right wrist (Figures 1A, B) showed patchy areas of lucency affecting the scaphoid and radial styloid. There was evidence of bony remodeling with well‐corticated margins affecting the radial articular surface, scaphoid waist, and dorsal aspects of the scaphoid bone, as well as evidence of mild radiocarpal joint osteoarthritis.

The magnetic resonance imaging (MRI) of the wrist revealed a periarticular multilobulated low T1 signal lesion with moderate post contrast enhancement wrapping around the dorsal and volar aspects of the proximal carpal row, essentially around the scaphoid and lunate. The mass lies deep to the dorsal radiocarpal ligament and is associated with splaying of the 3rd and 4th dorsal wrist compartment tendon sheaths. There was low signal of its capsule and intrinsic tiny focal areas of low signal septal nodularity reflective of hemosiderin deposits and no internal calcifications. There is associated well‐corticated bony erosions of the scaphoid waist, dorsal scaphoid body, and articular surface of the radial styloid with no aggressive periosteal reaction. Incidental high T2 bone marrow signal within the scaphoid, lunate, and underlying articulation is representative of bone marrow edema and is likely secondary to degenerative changes of the radiocarpal articulation (Figure 1C).

**FIGURE 2:**
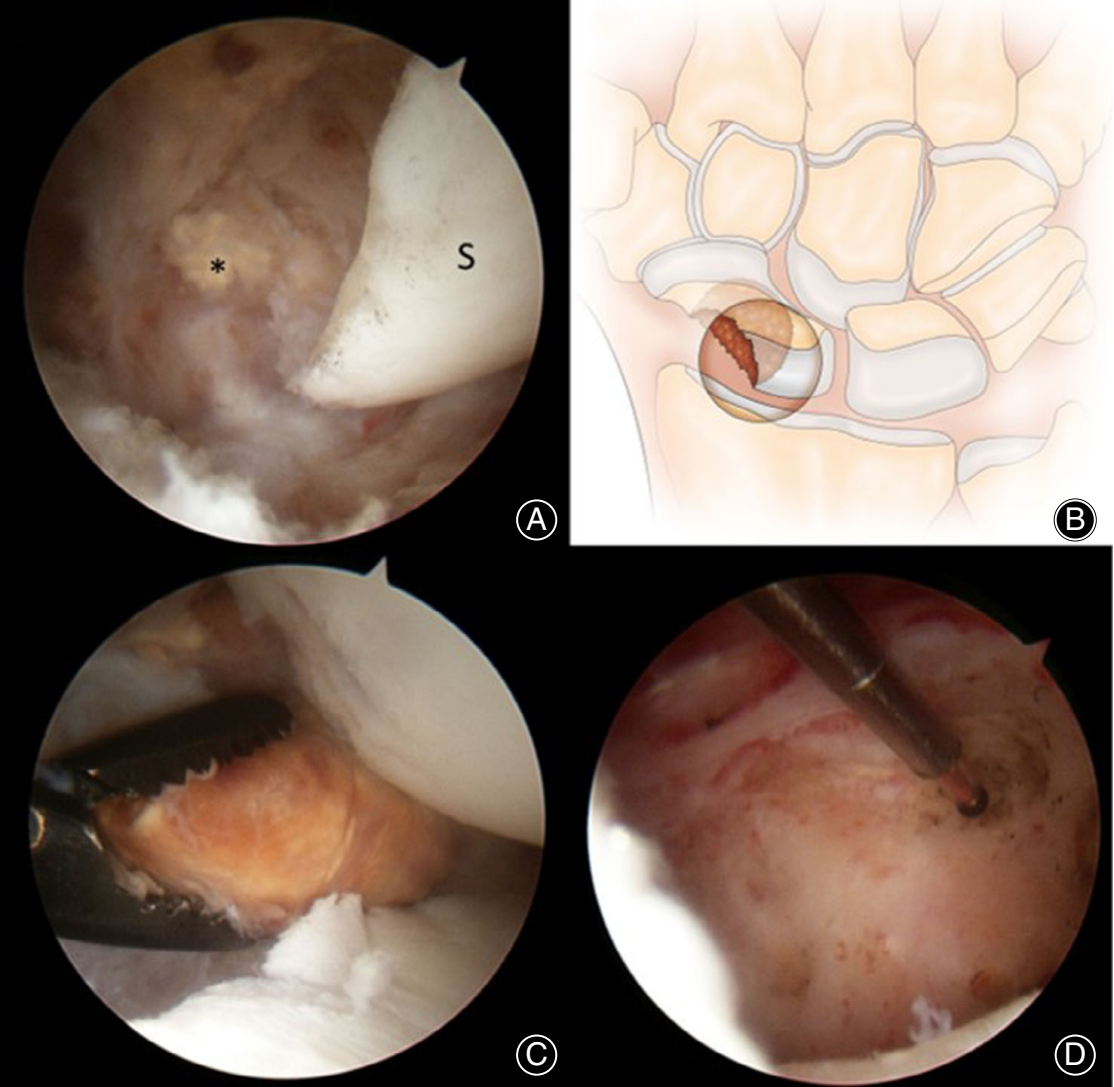
(A) Intraoperative wrist arthroscopy showing the direct visualization of the PVNS (*) invading into the scaphoid (S) with its characteristic fleshy yellow appearance, (B) Artist impression of the tumor eroding into the scaphoid with the circle depicting the arthroscopic view via the 3,4 portal. (C) Histological sampling performed arthroscopically using a grasper, (D) Radiofrequency ablation of tumor embedded within the scaphoid region.

The patient underwent arthroscopic evaluation of the wrist joint (Figure 2A, B), and histological sampling of the tumor was performed using a grasper (Figure 2C). The rest of it was excised piecemeal using a 3.5‐mm arthroscopic debrider and radiofrequency ablation was carried out for parts of the tumor embedded in the scaphoid (Figure 2D).

**FIGURE 3:**
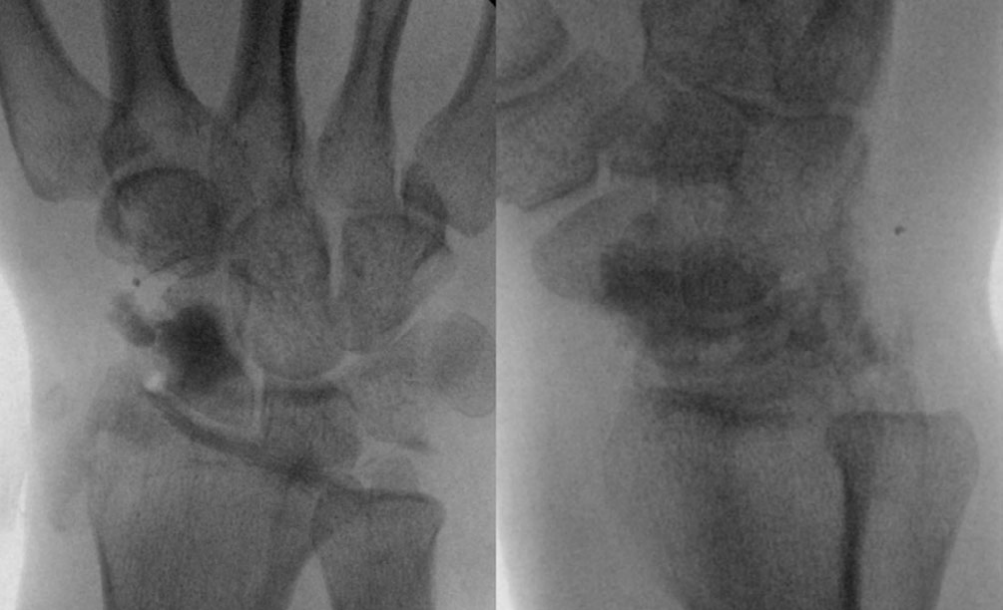
Fluoroscopy of wrist joint post artificial bone grafting (AP view), fluoroscopy of wrist joint post artificial bone grafting (lateral view).

The bony defect in the scaphoid was then filled with artificial bone grafting post resection and intraoperative fluoroscopy shows good filling of the defect (Figure 3).

**FIGURE 4:**
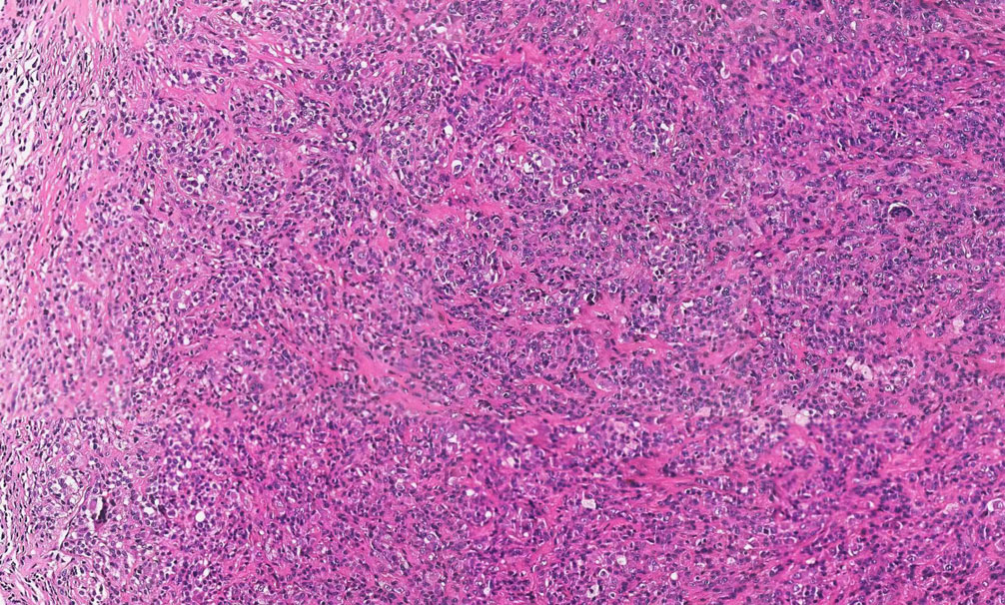
H&E staining of intraoperative histology confirming multinucleated giant cells.

Histological diagnosis confirmed the diagnosis of PVNS with no malignant features, showing monocytes, multinucleated giant cells, fibroblasts, and foam cells with cellular nodules separated by dense collagenous stroma (Figure 4).

Postoperatively, the patient was managed with a scaphoid splint for 4 weeks and hand therapy with periodic serial radiographic surveillance. His pain had largely resolved, and he was able to resume his duties as a dentist in after 1 month. His current follow‐up is at 5 months, and he remains pain‐free and satisfied.

## Discussion

PVNS is the second most common benign tumor of the hand with a plethora of names to describe it, including giant cell tumor of the tendon sheath, fibrous xanthoma, benign synovioma, and sclerosing haemangioma.^3^ Morphologically, they all comprise of synovial mononuclear cells and osteoclast‐like multinucleated giant cells.^1^ The occurrence of this tumor is deemed to be largely idiopathic^4^, ^5^ with surgical excision as the treatment of choice.

In terms of clinical presentation, there is some debate to whether pain was a distinguishing feature. In a study of 35 patients, most report a period of progressive pain, followed by diminished range of motion, and a possible mass effect.^1^ By contrast, a large case cohort study^3^ of 173 cases of PVNS showing a large female predominance (73%) with an initial presentation of a painless growing tumor (96%). Only two cases complained of tenderness likely due to proximity to a digital nerve. This corresponds to another large case cohort study^6^ of 216 patients with a male:female ratio of 1:2 with all of them having slow growing painless masses. For our patient, the escalating pain was likely due to progressive mass effect of the tumor onto the joint capsule and erosion of the scaphoid and radiocarpal joint.

The peak incidence of this tumor tends to occur in the 3^rd^–5^th^ decade of life in most studies,^7^, ^8^, ^9^ with one study reporting peak incidence to be around the 5^th^–6^th^ decade^2^ of life (25% and 27%, respectively). Our patient, in contrast, is of a younger age at 28. The usual location of these tumors tends to be in the hands primarily in the tendon sheaths of the index finger predominantly, followed by the middle finger and then the thumb.^7^, ^8^, ^10^ Previous studies report on average a 5.6% incidence in the wrist joint.^11^, ^12^, ^13^ In our patient it was in an unusual location of the radiocarpal joint with marked erosion into the scaphoid.

Although benign, it is locally aggressive with a high recurrence rate (7%–44%).^14^, ^15^, ^16^ Surgical excision is deemed to be the treatment of choice as this tumor is not known to resolve spontaneously.^6^ Table 1 is a literature review of two large cohort studies showing the treatment of PVNS located in the wrist, none of which were performed via arthroscopy. A large cohort study showed most of such recurrences occurring within 4 years of the index surgery, with a mean time of 25 months.^3^ This demonstrates the importance of complete excision with clean margins as well as continued surveillance after tumor excision. This is the first case report to the best of our knowledge, using wrist arthroscopic techniques to excise the tumor; we were able to perform meticulous resection under magnification with excellent visualization of the joint spaces and bony surfaces and ablate small areas of tumor embedded within the scaphoid with very fine instrument tips measuring 1.4 mm. The usual method of surgical treatment is that of the open technique, but this can cause dense adhesions and possible disruption to blood supply to the scaphoid and joint capsule. Wrist arthroscopy offers ideal visualization with a magnified view, is minimally invasive, and preserves the blood supply to the carpal region. Given the merits of wrist arthroscopy, we will advocate this method of surgical technique as an alternative for the trained hand in the treatment of PVNS of the wrist joint.

**TABLE 1:** Review of literature for treatment of PVNS in the wrist.

References	Number	Type of procedure performed	Complications of surgical resection	Recurrence time
Martin^1^	4	Open wrist synovectomy (3) Open surgical excision (1)	Data not available	No recurrence at average 54 months f/u (37–72 months)
Huang^6^	12	Open surgical excision (12)	Data not available	Data not available

Surgeons should be aware of the extent and location of such tumors as well as their anatomical relationships to critical structures such as nerves and vessels.^4^ This relationship will be best determined by preoperative imaging such as MRI with Wang et al.^17^ stating that MRI is the optimal modality of preoperative assessment of tumor size, extent and invasion of adjacent joint and tenosynovial space. PVNS should be on our list of differentials when we see bony concavity on X‐rays, other possibilities include intraosseous ganglion cysts or aneurysmal bone cysts. For our patient we obtained both X‐ray and MRI as part of our preoperative evaluation with wrist arthroscopy being employed intraoperatively to give direct visualization of the lesion which had the unmistakeable yellow fleshy multilobulated appearance of a giant cell tumor. The portals created also enabled us to fill the substantial bony defect of the scaphoid immediately post excision and ablation with artificial bone grafting.

The limitation of our paper is that it is on an experience of a single patient, but it brings to light an unusual presentation of painful PVNS in a seemingly rare location of the upper limb. We were able to excise the tumor and plug the bony defect through a minimally invasive procedure as opposed to the usual open techniques with good symptom resolution and return to work expediently. We are of the opinion that minimally invasive surgery can be considered as a method of excision for PVNS and mass effect of this tumor in joint spaces can lead to debilitating pain. The incisions are smaller yet through magnification of the sites of interest using wrist arthroscopy allows for thorough meticulous debridement of the tumor due to good visualization of the carpal anatomy. It leads to lesser scarring, helps in the protection of the blood supply to the wrist joint and surrounding structures as it is a minimally invasive procedure. The postoperative pain the patient experiences is also less and this aids in shorter hospital stays and faster postoperative rehabilitation. We do recognize that there is a learning curve to mastering this surgical technique, but the increased use of MIS procedures will be inevitable in the future.

## Author Contributions

All authors had full access to the data in the study and take responsibility for the integrity of the data and the accuracy of the data analysis. Conceptualization: L.B., Writing ‐ Original Draft: R.L, X.S.G, Writing‐ Review & Editing: R.L, L.B, Visualization: R.L, Study Supervision: L.B.

## Ethics Statement

The protocol for this research project has been approved by a suitably constituted Ethics Committee of the institution within which the work was undertaken and that it conforms to the provisions of the Declaration of Helsinki (as revised in Brazil in 2013).
